# Why Levallois? A Morphometric Comparison of Experimental ‘Preferential’ Levallois Flakes versus Debitage Flakes

**DOI:** 10.1371/journal.pone.0029273

**Published:** 2012-01-23

**Authors:** Metin I. Eren, Stephen J. Lycett

**Affiliations:** Department of Anthropology, University of Kent, Canterbury, Kent, United Kingdom; University College London, United Kingdom

## Abstract

**Background:**

Middle Palaeolithic stone artefacts referred to as ‘Levallois’ have caused considerable debate regarding issues of technological predetermination, cognition and linguistic capacities in extinct hominins. Their association with both Neanderthals and early modern humans has, in particular, fuelled such debate. Yet, controversy exists regarding the extent of ‘predetermination’ and ‘standardization’ in so-called ‘preferential Levallois flakes’ (PLFs).

**Methodology/Principal Findings:**

Using an experimental and morphometric approach, we assess the degree of standardization in PLFs compared to the flakes produced during their manufacture. PLFs possess specific properties that unite them robustly as a group or ‘category’ of flake. The properties that do so, relate most strongly to relative flake thicknesses across their surface area. PLFs also exhibit significantly less variability than the flakes generated during their production. Again, this is most evident in flake thickness variables. A further aim of our study was to assess whether the *particular* PLF attributes identified during our analyses can be related to current knowledge regarding flake functionality and utility.

**Conclusions/Significance:**

PLFs are standardized in such a manner that they may be considered ‘predetermined’ with regard to a specific set of properties that distinguishes them statistically from a majority of other flakes. Moreover, their attributes can be linked to factors that, based on current knowledge, are desirable features in flake tools (e.g. durability, capacity for retouch, and reduction of torque). As such, our results support the hypothesis that the lengthy, multi-phase, and hierarchically organized process of Levallois reduction was a deliberate, *engineered* strategy orientated toward specific goals. In turn, our results support suggestions that Levallois knapping relied on a cognitive capacity for long-term working memory. This is consistent with recent evidence suggesting that cognitive distinctions between later Pleistocene hominins such as the Neanderthals and anatomically modern humans were not as sharp as some scholars have previously suggested.

## Introduction

For over a century, archaeologists and palaeoanthropologists have been discussing a particular group of Palaeolithic flaked (i.e. knapped) stone cores and flake products that are collectively referred to as ‘Levallois’ [1], [2]. Named after the suburb of Paris (Levallois-Perret) from where they were recovered during the 19^th^ century, Levallois artefacts are now known to occur over large parts of Africa, western Asia as well as Europe [3]. In Africa, they appear to have a chronological origin ∼300 Kya [4], [5], and in Europe, Levallois is now also known to date from at least 300 Kya [6]. Indeed, the presence of Levallois artefacts is traditionally regarded as one of the main diagnostic features of the archaeological period referred to as the ‘Middle Palaeolithic’, or what in Africa is termed the ‘Middle Stone Age’ (MSA) [4], [5], [7]. With a wide geographic and temporal spread, the manufacturers of Levallois conservatively include at least three hominin species: *Homo sapiens*, *H. neanderthalensis* and late *H. heidelbergensis* (Archaic *H. sapiens sensu lato*) [8], [9]. The association of such artefacts with Neanderthals (e.g. [9], [10]) has, in particular, given rise to much debate regarding their potential significance for the evolution of hominin cognitive and linguistic capacities (e.g. [11], [12], [13], [14], [15]).

**Figure 1:**
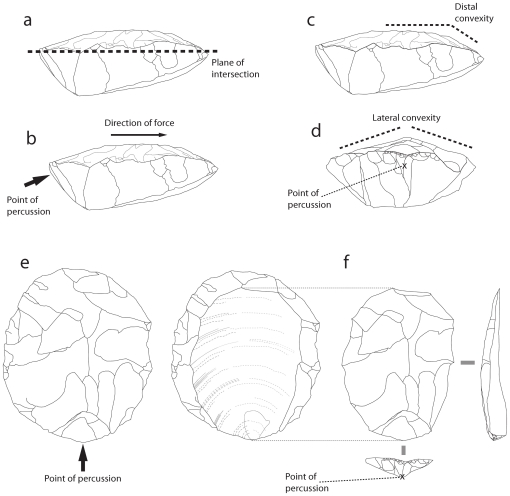
The ‘Volumetric’ concept of prepared Levallois cores. Two distinct surfaces intersect and define a ‘plane of intersection’ (a). Levallois flakes are removed parallel to the plane of intersection (b). Distal (c) and lateral (d) convexities determine the distal and lateral termination (i.e. flake margin) of the ‘preferential Levallois flake’ removed from the core via hard hammer percussion (e and f).

An important component in such debates relates to the fact that Levallois cores have frequently been thought to represent ‘prepared cores’. That is, the core is shaped in a deliberate manner such that the ‘Levallois flakes’ removed following such preparation are deliberately ‘pre-prepared’ and ‘predetermined’ in terms of overall size and shape [12], [16], [17]. Indeed, Levallois was once popularly identified and defined on the basis of specific flake products [18], [19]. More recently, however, ‘Levallois’ has more typically been identified on the basis of cores with specific properties of form and geometry [12], [20], [21], [22]. This ‘volumetric concept’ of Levallois (Figure 1) is based on six key criteria originally outlined by Boëda [23], [24], [25]: (1) the volume of the core is bifacial, comprised of two distinct surfaces that intersect at the core's margin, ultimately identifying a ‘plane of intersection’; (2) the two surfaces are organized hierarchically, whereby one surface is dedicated to the production of striking platforms that are used to detach flakes from the opposite ‘Levallois’ flaking surface; (3) the Levallois flake surface is shaped such that it possesses both distal and lateral convexities; (4) Levallois flakes are removed parallel to the plane of intersection; (5) the intersection (or ‘hinge’) of the striking platform surface and the flaking surface is perpendicular to the flaking axis of the Levallois flakes; (6) Levallois flakes are removed via direct hard hammer percussion. Although several of these stages may be achieved by a variety of different means, this volumetric concept has brought a level of coherence to Levallois such that cores identified as having been produced via this reduction processes exhibit a certain level of “homogeneity” ([26]: 201).

Despite the shift in emphasis away from flake products to cores and core reduction in the definition of Levallois, the concept of flake predetermination is still, however, inherent in Boëda's [25] ‘volumetric concept’, and conscious predetermination remains an important feature of Levallois according to many scholars (e.g. [12], [14], [17], [27], [28], [29]). This alleged predetermination has been used to support arguments for developed cognitive capacities in terms of foresight and ‘planning depth’ (e.g. [15]). Wynn and Coolidge [14] meanwhile have used Levallois to support arguments that extinct hominins such as Neanderthals possessed a long-term working memory, which allows the rapid retrieval of knowledge from long-term memory thus enabling ‘expert’ levels of performance. Notions of predetermination in Levallois have also been used to support arguments relating to linguistic capacities in extinct hominins. For instance, some time ago Holloway ([30]: 403) (in conceiving of Levallois production as a structured goal-orientated activity) suggested that “as in language, the activity is made up of units concatenated non-randomly, there being contingencies both in language pattern and tool-making” such that there is a “grammar” involved in both activities. With regard to interconnecting concepts made up of minimal unit activities, he went onto state ([30]: 404) that “the alphabet of chipping technique is not random either … where certain of these are contingent upon prior operations (e.g. Levallois technique)”. Lieberman ([11]: 163–170) also drew on concepts of Chomskian grammar to link the processes of Levallois reduction with the cognitive processes involved in language (although see [31]: 257).

All such arguments pertaining to the cognitive and linguistic capacities of extinct hominins (regardless of other details relevant to their merit) are obviously contingent upon the premise that production of ‘Levallois flakes’ is a deliberate, goal-orientated activity, predicated around the production of ‘preferred’ and ‘predetermined’ flakes. However, not all accept that Levallois flaking involves strong concepts of predetermination or conscious, structured planning. For instance, Noble and Davidson ([13]: 200) suggest that the alleged phases of extensive preparatory flaking involved in ‘Levallois’ flake production implies “a wastefulness of knapping effort and raw material that seems implausible”. Likewise, Sangathe ([32]: 148) has argued that since “most flakes produced with skill and regularity … have sharp usable edges … it does not seem likely that the advantage acquired by producing a flake of specific shape was sufficient to necessitate the extra effort required by employing the Levallois technique”. In the absence of predetermination, it has been argued, the “time depth of intentionality is reduced to decisions about the next flake, and not to decisions about the final form” ([33]: 376). Rejection of notions concerning predetermination or planning in Levallois industries has therefore led some to suggest that not until the Upper Palaeolithic do we see clear “marks of planning that seem to entail a capacity for consciousness” ([33]: 382).

Suspicions regarding the ‘preferred’ and ‘planned’ nature of Levallois flakes led Sangathe [32] to the novel suggestion that removal of large central flakes was primarily a core maintenance strategy intended to reduce the central mass of a core allowing the establishment of a consistent core morphology throughout reduction. Importantly, Sangathe ([32]: 157) recommended that experimental flintknapping could be used to test his proposition. However, subsequent experiments by workers following this recommendation failed to support the central tenets of his hypothesis, and demonstrated that a consistent core morphology is readily maintained in the absence of Levallois removals [34].

Examinations of archaeological material that might shed light on the issue of Levallois predetermination have produced mixed results. In one of the most comprehensive studies of the issue, Dibble [35] examined flakes from 27 different assemblages in southern France. Specifically, he focused on the issue of predetermination in Levallois flakes and the allied notion that certain flakes were more desirable than others, such that their production could be linked to language categories ([35]: 424). The logic underlying his analysis was that if by ‘predetermination’ a level of standardization was implied, then it can reasonably be expected that there will be less variability in Levallois flakes compared with other flake categories. Flakes from each assemblage were divided into three categories: Levallois flakes, biface retouch flakes, and indeterminate ‘normal’ flakes. An analysis of flake area, length, width and thickness measurements (and their ratios) suggested that Levallois flakes were not necessarily statistically more standardized, thus leading Dibble ([35]: 425) to argue that their manufacture could not be linked to “the presence of linguistic rules, structures, or categories”. A study by Schlanger [12], however, used flakes from a refitted Levallois core from the Middle Palaeolithic site of Maastricht-Belvédère (Netherlands) and reached a different conclusion. Here, he found that length, widths and thicknesses of the nine Levallois flakes were, as a group, more standardized than the 32 non-Levallois (debitage) flakes.

Working within the restrictions automatically imposed whenever dealing with Palaeolithic archaeological materials, these previous studies inevitably possess certain weaknesses alongside particular strengths in each case. The major strength of Dibble's [35] study was a large overall sample size. However, there is an inevitable degree of subjectivity in assigning flakes to different categories (i.e. ‘Levallois’, ‘biface’, etc.) in the absence of additional information, such as might be obtained through refitting. Indeed, controlled experiments have demonstrated that even in the case of experienced workers, the accurate identification of Levallois flakes over other categories of flake is subjective and non-replicable across participants [36]. Moreover, Schlanger ([12]: 249) pointed out an apparent incongruity in Dibble's [35] study, whereby the categorization of certain flakes as ‘Levallois’ was achieved in the initial phases, yet the subsequent quantitative analysis did not indicate standardization. Similarly, Kuhn [37] has noted that selecting flakes from a range of varying archaeological examples and classifying them on the basis of certain properties (e.g. as ‘Levallois’, ‘biface flake’, etc.) might inevitably lead to them being regarded as ‘standardized’ in a subsequent metric analysis. In using a refitted core, Schlanger [12] was on a somewhat firmer, although not entirely assumption free, basis with regard to classifying certain flakes as ‘Levallois’. However, in using a sample size of just 41 total flakes from a single (incomplete) core, statistical validity is open to question since inferential statistical methods were not applied. In addition to these points, it is notable that both of these studies used simple measurement schemes (essentially three primary measurements of length, width and thickness) and neither study utilized multivariate statistical approaches. While the use of relatively simple morphometric methodologies alone does not necessarily negate the various arguments concerning standardization and ‘preference’ in Levallois flakes, it does mean that only limited aspects of flake variability were examined in these previous studies.

Clearly, in light of the foregoing, a level of ambiguity concerning the ‘predetermined’ nature of Levallois flakes is evident. Here, therefore, we adopted an experimental approach to the issue. We focus on the production of ‘classic’ lineal or so-called ‘preferential’ Levallois (‘tortoise’) cores and their products ([12]: 238, [22]: 65, [25]: 56), which have figured prominently in the issues discussed previously. The use of experimental assemblages allows us to negate the problems associated with arbitrarily assigning archaeological flakes to different categories. It also enabled the generation of flake samples large enough (*n* = 642 flakes) to be amenable to several inferential statistical analyses. In addition, we used a morphometric scheme involving 15 size-adjusted variables, thus enabling multivariate methodologies to be applied, and issues of flake size and shape to be disentangled more directly during analysis. Prior to our main analyses of the flakes, we also established (via a comparative analysis) that the experimental cores produced in our experiments replicated known archaeological examples of Levallois core accurately.

Our analyses focused on two issues. Firstly, if so-called ‘preferential’ Levallois flakes (hereafter putative PLFs) produced on classic ‘tortoise’ cores were genuinely a ‘preferred’ product with common properties uniting them as a coherent entity or ‘category’ of flake, then they should possess a series of particular attributes that identify them as a group more consistently than the debitage flakes produced during their manufacture. Accordingly, we tested this prediction using size-adjusted morphometric data and the multivariate statistical technique of discriminant function analysis. Secondly, if PLFs produced through tortoise ‘Levallois’ core reduction represent genuinely ‘preferred’ products engineered (via this volumetric core reduction strategy) to meet specific requirements, they should possess a greater standardization in their attributes compared with the debitage flakes produced during their manufacture. We tested this prediction using coefficients of variation for each of the attributes. Moreover, in both cases, we aimed to identify *which particular* attributes might unite PLFs as a coherent entity, or in the case of standardization, *which particular* attributes, appear to be relatively more standardized in PLFs as opposed to the flakes produced during their manufacture. Our rationale here was that if particular attributes unite PLFs as a consistent and coherent flake group and the volumetric construction of the core results in them being controlled (i.e. ‘standardized’) in a particular manner, then it should be possible to relate these variables to current archaeological knowledge concerning the functionality and practical desirability of certain flake forms over others. In other words, our analyses aimed to establish on a more firm basis *why* Levallois flakes might have been a preferred and targeted product during Levallois reduction; an issue to which we turn in our discussion.

## Materials and Methods

### Knapping the Levallois reductions

One of us (MIE) knapped a total of 75 PLFs from a series of 25 nodules of Texas chert from the Cretaceous-aged Fredericksberg Group [38]. The number of PLFs produced from each nodule ranged from 1–5 (mean = 3). Each Levallois reduction was specifically configured to conform to Boëda's [25] criteria for Levallois, via the production of a classic lineal ‘preferential’ (tortoise) Levallois core. Following Bradley ([39]: 22), Levallois reduction was comprised of two stages using direct hard hammer percussion throughout. The first stage establishes the preliminary bifacial margin, which is continuous around the circumference of the nodule. Stage two, involves three sub-stages: (1) shaping of the Levallois flaking surface and margin adjustment; (2) preparation of the PLF platform; (3) removal of PLFs.

Again, following Bradley [39], we defined ‘ventral’ flakes as those removed from the face from which the putative PLFs are removed, and refer to flakes removed from the non-PLF surface as ‘dorsal’ flakes. This is potentially confusing as Levallois cores are typically illustrated with the Levallois flaking surface facing upward (i.e. superiorly). However, it should be noted that when the putative PLFs are eventually removed from the core, it is orientated such that the Levallois surface is facing downward (i.e. ventrally), thus establishing the terminology used here. For each Levallois reduction, all debitage flakes from the dorsal and ventral surfaces were bagged separately and labeled. Each PLF was also bagged and labeled. Following this cataloging procedure, all subsequent stages of sampling, data recording, and analysis were performed by SJL thus ensuring an independence between the knapping and data analysis phases of the study.

The manufacture of Levallois products is generally considered a highly skilled activity and it has been claimed that only a relatively limited number of contemporary knappers are able to produce replications that stand close scrutiny alongside archaeological examples ([14]: 474, [15]: 118). Hence, a comparative 3D geometric morphometric analysis of the experimental cores resulting from the production of flakes used in our later analyses was also undertaken (Text S1). This analysis demonstrated that the replica cores fit comfortably within the range of variation exhibited by a sample of genuine archaeological examples of 152 Levallois cores found at sites in Africa, western Asia and Europe (Text S1, Figure S1, Figure S2, Figure S3). Importantly, this thus verifies quantitatively that Levallois core morphologies were replicated with high degrees of accuracy compared with known archaeological examples.

### Flake sampling protocol

A total of 642 experimentally produced flakes were examined in this study, including the 75 ‘Preferential’ Levallois flakes. There is some evidence to suggest that wherever a range of flake sizes are available, extremely small flakes (i.e. <2 cm in length) would less likely have been utilized as hand/finger held tools [40]. Moreover, in the context of the current analyses, extremely small flakes/chips are, *a priori*, those least likely to share form affinities with PLFs. Therefore, only debitage flakes >2 cm in maximum length were measured. A maximum of eight complete debitage flakes per PLF were measured; up to four from the PLF (ventral) surface and up to four from the non-PLF (dorsal) surface. Wherever the total number of potentially measurable flakes from a surface exceeded four specimens, four flakes were sampled randomly using a random number generator (http://www.randomizer.org). Application of this strategy resulted in a total of 567 debitage flakes being compared against the 75 putative PLFs.

### Flake attributes

A total of 15 quantitative variables were measured for all flakes and are listed in Table 1. Full details and descriptions of these measurements can obtained in the supporting information (Text S2).

**Table 1:** List of variables measured for each flake analysed (full descriptions available in S2).

1	Maximum length
2	Maximum width
3	Width at 25% of Maximum length
4	Width at 50% of Maximum length
5	Width at 75% of Maximum length
6	Length of flake (technological)
7	Thickness at 25% of Maximum length
8	Thickness at 50% of Maximum length
9	Thickness at 75% of Maximum length
10	Thickness at 25% of Maximum width
11	Thickness at 75% of Maximum width
12	Maximum flake thickness
13	Bulb thickness
14	Length of sharp edge
15	Index of symmetry

### Analysis 1: Discriminant analysis of flake attributes

If PLFs were genuinely a ‘preferred’ product with common properties that unite them as a coherent entity or ‘category’ of flake, then they should possess a series of attributes that identify them as a group more consistently than the debitage flakes produced during their manufacture.

Such a prediction may be tested multivariately using Discriminant Function Analysis (DFA). Analytically, DFA provides a set of weightings (i.e. discriminant functions) that most effectively discriminate between groups that are defined a priori [41], [42]. Such weightings are linear combinations of the original variables. The relative coherency of specific groups (in terms of the original variables) may be assessed by the extent to which individual specimens can be classified back to their original group, with results frequently expressed in percentages (%). Importantly, the DFA also identifies which of the attributes are most important in assigning specimens to groups. Here, the DFA was undertaken in SPSS v16.0. Conservatively, only cross-validated results were examined, whereby specimens are classified in turn on the basis of linear functions derived from all other specimens except that specific case [42].

If PLFs are genuinely a specific category, with common properties that unite them as a group with relatively high degrees of consistency, it may in this specific instance be predicted that in a DFA of PLF, dorsal and ventral flake groups, PLFs will be classified more accurately than either dorsal or ventral flakes, and with a relatively high degree of accuracy. The ratio of correct to incorrect classifications for each flake group may be assessed for statistical significance (α = 0.05) relative to chance (*H_0_* = 50∶50) using a chi-square (χ^2^) test. Note here that in the original DFA, the probability of a flake being assigned to its correct group by chance alone is 33.3%. However, since the χ^2^ test is simply asking whether the chance of a flake being classified correctly in the original DFA is significantly different from the chance of it being misclassified (i.e. in cases of misclassification the test is not taking into account which of the other two groups it has been assigned to), chance in this latter instance is 50%.

Given that PLFs will on average be bigger than many debitage flakes in a Levallois reduction sequence, all data were size-adjusted in order to analyse their shape properties as opposed to merely examining size differences. Moreover, by size-adjusting the data this ensures that results will be generally comparable across tortoise Levallois cores, regardless of overall size, which may be especially important given that archaeological examples of Levallois cores vary greatly in isometric size [43]. Attributes 1–13 were size adjusted by the geometric mean of those measurements [44], [45], and attribute 14 (length edge of sharp edge) was size-adjusted using the geometric mean of all plan-form variables (i.e. attributes 1–6). The Index of Symmetry is a scale-free variable (Text S2) and was inputted to the DFA directly.

### Analysis 2: comparison of standardization

If PLFs are genuinely ‘preferred’ products engineered to meet specific requirements, they should possess a greater standardization in their attributes compared with the debitage flakes produced during their manufacture. Following Dibble [35] and Schlanger [12] relative standardization in the attributes of PLFs compared with debitage flakes may be assessed directly through comparison of coefficients of variation (CV) of the raw measurements expressed as percentages (i.e. standard deviation/mean×100). Hence, in order to test predictions of standardization a CV was calculated for each attribute. Thereafter, the overall extent of standardization in PLFs versus debitage flakes was assessed for statistical significance via a Mann-Whitney U-test (α = 0.05) of the two groups of CV values. Because the Index of Symmetry is a scale-free variable (Text S2), descriptive statistics such as means and standard deviations may be compared across flake groups directly. Therefore, in this instance, the difference in flake symmetry across groups was assessed using a Mann-Whitney U-test, while an F-test was used to determine differences in the standard deviation of each group (α = 0.05).

## Results

### Analysis 1: Discriminant function analysis of flake attributes

**Figure 2:**
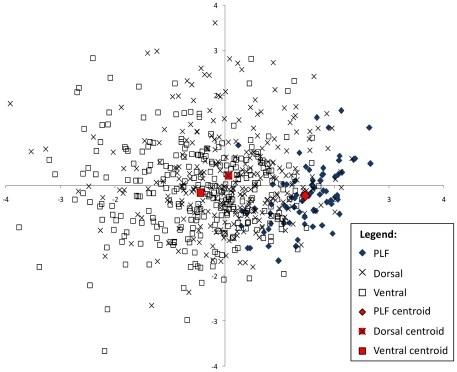
Plot of discriminant functions 1 (x axis) and 2 (y axis) resulting from the DFA (see **Table 2** for classification scores).

Figure 2 shows the plot of the DFA scores (functions 1 and 2) for the 642 flakes. Function 1 explained 90.1% of variance and is statistically significant (Wilks' Lambda = 0.715; p<0.0001). As Table 2 shows, PLFs were correctly classified to group in 89.3% (cross-validated) of cases, well over twice as high (i.e. 2.682×) as would be predicted by chance alone (33.3%). Conversely, dorsal debitage flakes were correctly classified to group in only 36.7% of cases, while ventral flakes could be classified to their correct group in only 54.3% of cases. Dorsal and ventral debitage flakes were consistently misclassified with each other to a greater extent than they were as PLFs (Table 2). The ratio of correct to incorrect classifications for PLFs was significantly greater than chance (χ^2^ = 60.840; df = 1; exact p<0.0001). In the case of dorsal flakes, the ratio of correct to incorrect classifications was significantly below chance (χ^2^ = 6.760; df = 1; exact p = 0.012). For ventral flakes, the ratio of correct to incorrect classifications was not significantly different from chance (χ^2^ = 0.640; df = 1; exact p = 0.484). Hence, the results of the DFA support the hypothesis that the PLFs (as a category of flake) share a particular combination of attributes, robustly identifying them as a coherent group.

**Table 2:** Results of Discriminant Function Analysis.

Classification by *n* (cross validated)	PLF	Dorsal	Ventral	Total (*n*)
**PLF**	**67**	5	3	75
**Dorsal**	72	**102**	104	278
**Ventral**	36	96	**157**	289
**Classification by % (cross validated)**	PLF	Dorsal	Ventral	Total (*n*)
**PLF**	**89.3**	6.7	4.0	75
**Dorsal**	25.9	**36.7**	37.4	278
**Ventral**	12.5	33.2	**54.3**	289

It is also notable that the variables loading most highly (positively) on DF1 and thus contributing to the positioning of the PLFs on that function (and their classification rate) were the five flake thickness variables (i.e. Thickness at 25, 50 and 75% of Length, and Thickness at 25 and 75% of Maximum Width). This suggests that control of these thickness variables was an important feature of PLFs.

### Analysis 2: comparison of standardization

CVs for debitage flakes were consistently higher for all variables (Table 3). Differences between the two groups of CVs for PLF versus debitage flakes were statistically significant (Mann-Whitney U = 48.0; asymptotic p = 0.022; exact p = 0.021). Likewise, mean symmetry measures and their standard deviations were higher for debitage flakes than for PLFs, thus demonstrating that PLFs are, on average, more symmetrical and exhibit less variability in this attribute. Differences between flake categories were statistically significant for overall symmetry measures (Mann-Whitney U = 11227.0; asymptotic p<0.0001) and for their standard deviations (F = 37.108; d.f. = 1; p<0.0001). Hence, the results of this analysis consistently support the hypothesis that PLFs are more standardized in form than debitage flakes.

**Table 3:** Results of CV analysis and descriptive statistics.

	Mean (mm)		SD		CV (%)^1^		CV difference
Attribute	PLF	Debitage	PLF	Debitage	PLF	Debitage	
Maximum length	82.32	52.79	21.18	15.06	25.73	28.54	2.81
Maximum width	61.04	35.41	15.64	11.69	25.62	33.02	7.4
Width at 25% of Max length	46.69	23.20	11.84	8.84	25.36	38.10	12.74
Width at 50% of Max length	59.00	32.36	15.23	11.05	25.82	34.16	8.34
Width at 75% of Max length	53.75	30.98	14.36	10.51	26.73	33.92	7.19
Length of flake (technological)	79.22	44.95	21.89	15.22	27.62	33.87	6.25
Thickness at 25% of Max length	9.45	3.30	4.71	2.78	49.87	84.14	34.27
Thickness at 50% of Max length	12.51	4.86	5.67	3.67	45.31	75.47	30.16
Thickness at 75% of Max length	12.68	5.14	5.53	3.85	43.59	74.95	31.36
Thickness at 25% of Max width	9.84	4.26	4.66	3.36	47.40	78.79	31.39
Thickness at 75% of Max width	9.73	4.17	4.53	3.29	46.57	79.00	32.43
Maximum flake thickness	15.32	7.74	6.00	4.45	39.15	57.51	18.36
Bulb thickness	13.79	6.55	5.18	3.91	37.60	59.71	22.11
Length of sharp edge	185.53	106.75	51.21	36.33	27.60	34.03	6.43
Index of symmetry	0.40	0.73	0.25	0.46	–	–	–

1 Overall Mean CV values (%) are 35.28 for PLFs and 53.23 for debitage flakes.

Consistent with the results of the DFA analysis, it should also be noted that the attributes with the highest differences in CV values between debitage flakes and PLFs were the five thickness measurements of debitage flakes along the various percentage points of maximum length and maximum width (Table 3). Again, this suggests that PLFs (relative to alternative flake categories) are a means of engineering consistency of flake thickness within specific bounds, across a large proportion of their surface area.

## Discussion

In our first (DFA) analysis, dorsal flakes were correctly classified to group at levels barely above chance, and in the case of ventral flakes, almost every other flake was misclassified. Conversely, in the case of PLFs, only around one in ten flakes were misclassified. Most importantly, only in the case of PLFs was the ratio of correct to incorrect classifications statistically greater than chance. Hence, in line with the hypothesis that PLFs are a ‘preferred’ product with common properties that unite them as a coherent entity, this first analysis demonstrated that PLFs form a robust group with a relatively consistent relationship between measured variables. It is also notable that the most important variables driving their performance in the DFA were measurements relating to relative flake thickness.

In the second analysis, it was found that the PLFs were significantly less variable than the debitage flakes produced during their manufacture. PLFs were also found, on average, to be significantly more symmetrical than debitage flakes. Importantly, in a manner consistent with the results of the first analysis, the greatest differences between CV values for PLFs versus debitage flakes were observed in the five variables measuring flake thickness along their maximum lengths and widths. This is despite the fact that maximum thickness was found to be more variable than maximum length or width measures in both PLFs and debitage flakes. The results of this multi-core analysis are thus consistent with Schlanger's [12] examination of flakes from a single archaeological Levallois core, in terms of showing that maximum thickness is more variable than maximum length or width measures (regardless of flake category), but more importantly, in corroborating his assertion that PLFs exhibit less overall variability than the debitage flakes removed during their production. Moreover, in this instance, the statistical significance of this distinction has been established.

Overall, therefore, the results of our analyses demonstrate that PLFs form a relatively coherent entity with a set of specific properties that unite them robustly as a group or ‘category’ of flake. The properties that do so, relate most strongly to relative flake thicknesses across the surface area of PLFs. In addition, our analyses demonstrate that PLFs exhibit significantly less variability than the flakes generated during their production, and that such relative standardization is again most evident in variables relating to flake thicknesses across the length and width of PLFs. Hence, our results are consistent with propositions (e.g. [12], [14], [28], [29]) that Levallois flakes are standardized in such a manner that they may be considered ‘predetermined’ with regard to a specific set of properties, even when adjusted for overall size differences.

A further specific aim of our study was to determine whether the *particular* PLF attributes identified during the course of our analyses, can be related to existing archaeological knowledge concerning the potential functionality and practical desirability (i.e. utility) of certain flake forms over others. In other words, do our analyses provide further insight into *why* Levallois flakes manufactured on classic ‘tortoise’ cores might logically have been a ‘preferred’ product having been standardized in such a manner?

Mobility is a factor in the lives of all hunter-gatherer populations, although the extent and pattern of such mobility may vary greatly ([46]: 111–160, [47]). Transport distances of lithic raw materials appear to increase during the course of the European Middle Palaeolithic, suggestive of increased mobility [48], [49], [50], with similar evidence available for the African MSA [51]. Such evidence has led to suggestions that Levallois was a technology geared specifically toward increased mobility [52]. Regardless of this, given that Pleistocene hominins were foragers, mobility was inevitably a feature of their existence. As Kuhn ([53]: 427) has noted “mobile toolkits should tend to optimize their potential usefulness relative to weight, the primary determinate of transport cost”. Moreover, such artefacts “should be durable and inherently ‘maintainable’” ([53]: 428). From the viewpoint of optimality, therefore, the most ideal flake cutting tool is one that provides the greatest utility/durability relative to transport cost (i.e. weight).

Modeling the potential utility of different flake sizes, Kuhn ([53]: 430–432) has shown that potential for retouch (i.e. resharpening) is directly proportional to increased flake area, although the relative increase in utility (so defined) diminishes as flake area increases (i.e. as flakes become heavier). Moreover, under the assumptions of such a model he has shown that decreasing the relative thickness of a flake increases its retouch potential relative to mass ([53]: 432). A further adjustment to the model showed that if the increased amount of cutting edge provided on larger tools was accounted for, utility declines relative to increasing mass as before, but that the rate of relative decline decreases under these conditions ([53]: 435).

The large surface area of PLFs compared to flakes from the same core is a feature that was noted in some of the earliest commentaries on Levallois ([1]: 225), and has been repeated on many occasions since (e.g. [12]: 241, [32]: 148). This is also clearly evident in our results given the mean lengths and widths of PLFs compared to debitage flakes (Table 3). PLFs removed from tortoise cores would, therefore, appear to provide a relatively large potential for retouch under the parameters of Kuhn's [53] model. However, as Kuhn ([53]: 430) himself notes, the model does not assume that differing flake thicknesses might directly impact utility (however measured), nor does the model account for the fact that flake weight itself may have functional advantages affecting optimization factors. When applying a flake tool to a task, greater force may be applied either by the tool-user exerting greater pressure [54], [55], or by choosing relatively heavier tools such that gravity increases momentum. Indeed, experiments have shown that larger flake cutting tools exhibit greater cutting efficiency than smaller flakes [40]. This suggests that alongside Kuhn's [53] observations regarding utility in terms of retouch potential, the fact that Levallois flaking enables the production of large flakes (relative to the size of the core) would also provide an advantage in terms of cutting efficiency, at least compared to debitage flakes from the same core.

However, these factors aside, the strongest patterns emerging from our analyses were related to the thicknesses of PLFs, both in terms of classification and standardization. Examination of Table 4, which shows the averages for flake thickness measurements in the size adjusted data, gives greater insight into the precise parameters underlying this statistically significant pattern. Four factors are evident in this Table. Firstly, PLFs (relative to size) are on average thicker across their surface area (as a whole) than debitage flakes. Secondly, maximum thickness in PLFs (relative to size) is less for the PLFs than for the debitage flakes. Thirdly, examination of the six individual thickness measurements shows that thickness is greater (relative to size) in PLFs for all thickness measurements except for maximum thickness, indicating that maximum thickness is reduced relative to the other measurements, and contributing to the relatively *even* thickness of PLFs throughout their surface area. Fourthly, PLFs are less variable across all thickness measurements (i.e. thickness is more evenly distributed, as indicated by the lower standard deviation of the means).

**Table 4:** Summary data for size-adjusted thickness data.

	Thicknesses along length			Thicknesses along width		Maximum Thickness		
	Mean Thickness at 25% (size adjusted)	Mean Thickness at 50% (size adjusted)	Mean Thickness at 75% (size adjusted)	Mean Thickness at 25% (size adjusted)	Mean Thickness at 75% (size adjusted)	(Size adjusted)	Mean of the mean thickness variables (size adjusted)	SD of the mean thickness variables (size adjusted)
**PLFS**	0.371	0.483	0.498	0.384	0.380	0.607	0.454	0.093
**Debitage**	0.269	0.387	0.417	0.344	0.335	0.647	0.400	0.131

**Figure 3:**
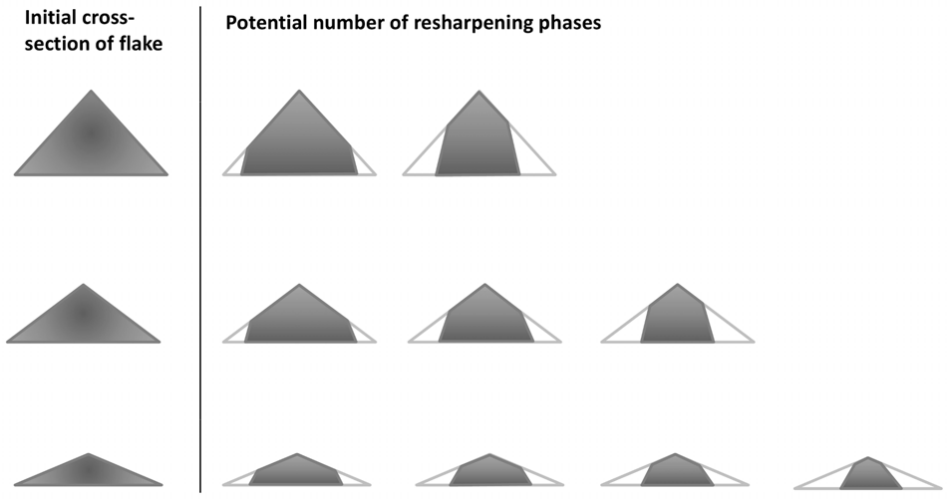
Schematic representation of the relationship between the original cross-section of a flake (i.e. evenness of thickness across surface area) and the total potential number of instances of resharpening (redrawn and modified after Turq, 1992).

These factors may be related directly to several different utility/efficiency issues. As noted, for simplicity, Kuhn's [53] model assumed that flake thickness did not affect utility, and suggested that increasing flake area equated to increased retouch potential. At the same time, his model suggested that reducing flake thickness would reduce weight without reducing utility (i.e. retouch capacity). However, thin flakes also break more easily ([56]: 150). Hence, a flake so thin that it disintegrates upon usage and/or retouching would negate any advantage of large flake size (i.e. plan-view surface area), and it is now recognized that edge durability (i.e. the capacity to withstand attrition upon use) is a factor that would have affected hominin decision making in factors relating to cutting tools ([57], [58]: 1612). Even a flake with only a portion of its surface area that is too thin to provide a viable working edge, would exhibit reduced utility relative to its absolute surface area. The relative thickness distributed evenly across PLFs would, therefore, provide support for a viable and robust working edge across the greatest extent of its surface area. Moreover, the fact that maximum thickness in PLFs does not appear to increase proportionally with regard to the other thickness variables, indicates that carrying-weight is reduced directly in the portion of flake area that is typically the least utilizable in flakes (see e.g. [53]). As Turq ([59]: 77) has shown, flakes with a more evenly distributed thickness of cross-section themselves have a greater potential for retouch and re-use (Figure 3).

In addition to these points, our results indicate that several factors relating to ergonomic considerations and efficiency during use may also have made PLFs desirable relative to other flakes. For instance, increased relative symmetry in a cutting tool, and an evenly distributed thickness, “puts the center-of mass of the tool in the line corresponding to the direction of motion of the tool at the instant of impact, thus avoiding torque and, consequently, maximizing power” (i.e. efficiency) [60]. Moreover, experiments with handaxes have shown empirically that there is a statistically significant relationship between increased symmetry and increased efficiency in cutting performance [61]. An increased regularity of surface would “distribute the reaction force at impact time more evenly through the hand of the tool's user, which increases comfort” [60]. These proposed advantages of PLFs are not, of course, contingent upon a presupposition that debitage flakes were not utilized, nor are they mutually exclusive to suggestions that the volumetric reduction strategy of Levallois is itself an economic means of reducing cores and maximizing productivity [21]. Indeed, the multiple potential reasons for the utilitarian advantages of Levallois would explain its manufacture by at least three different hominin species and its widespread geographic distribution.

As some have noted, all flakes removed from a core are to some extent influenced by the morphology of the core (angle, curvature, flake scar pattern, etc.) prior to their detachment ([12]: 235, [22]: 63). A flake bearing the scars of previous removals is, therefore, both automatically ‘predetermined’ and *predetermining* with regard to any future removals. What our results suggest, however, is that predetermination via the multi-phase volumetric construction of a Levallois/tortoise core (*sensu* [25]) enables this predetermination of PLFs to be *engineered* in a particular way that ultimately (and significantly) distinguishes PLFs from a majority of other flakes. Moreover, those *particular* attributes may be linked to certain specific factors that, based on current knowledge, can be suggested as potentially desirable features when faced with a choice of alternative flake forms. Future experiments are now required to more accurately model the advantages of PLFs over alternative categories of flake. It should also be emphasized that our analyses have focused on ‘classic’ lineal Levallois, and that further experiments should explore alternative forms of core incorporated under the term ‘Levallois’. Indeed, some experiments have shown previously that ‘point’ Levallois flakes may have functioned as projectile points [62], [63].

In suggesting that Levallois flakes were indeed a genuinely predetermined and preferred product, our results also have implications for the cognitive and linguistic debates associated with Levallois. That is, our results are consistent with the hypothesis that the execution of Levallois knapping is evidence of an ability to draw on a cognitive capacity of long-term working memory [14]. Direct evidence for language is perhaps unlikely to come from stone tools, and such evidence should always be supplemented with anatomical and palaeoneurological evidence (e.g. [64], [65]). However, our results are consistent with analogies between the hierarchical structuring of information such that it results in a specific goal, and the hierarchical organization of syntax and grammar (e.g. [66]: 129) in sentence construction [11], [30], and suggest that such analogies and their implications are worthy of future exploration. Moreover, our results also suggest that Middle-Late Pleistocene hominins attributed to *H. heidelbergensis* (*sensu lato*), *H. neanderthalensis* and early *H. sapiens* were all, at least on occasion, solving problems associated with lithic resource optimization and the optimization of flake tool technology in the same manner (i.e. via Levallois). Our results are, therefore, consistent with recent evidence (e.g. [67], [68]) suggesting that cognitive capacities in different species of Middle-Late Pleistocene hominins are not as sharply differentiated as previous generations of scholars postulated, and that the behavioural changes that eventually emerge during the Later Stone Age (African LSA) and Upper Palaeolithic may be more the product of demographic change and increased connectivity of social networks [69] than they were, necessarily, of fundamental cognitive changes.

## Supporting Information

Text S1 **A comparative 3D geometric morphometric analysis of the experimental Levallois cores and archaeological examples.**
(DOC)

Text S2 **Flake variables measured for analyses.**
(DOC)

Figure S1 **Principal component results of 3D geometric morphometric analysis comparing archaeological examples of Levallois core against the experimental replicas produced for this study.** This Figure shows PC1 plotted against PC2.
(TIF)

Figure S2 **Principal component results of 3D geometric morphometric analysis comparing archaeological examples of Levallois core against the experimental replicas produced for this study.** This Figure shows PC1 plotted against PC3.
(TIF)

Figure S3 **Principal component results of 3D geometric morphometric analysis comparing archaeological examples of Levallois core against the experimental replicas produced for this study.** This Figure shows PC2 plotted against PC3.
(TIF)

## References

[1] De Mortillet G (1883). La Préhistoire: Antiquité de l'Homme.

[2] Commont V (1909). L'industrie moustérianne dans la région du Nord de la France. Congès Préhistorique de France 5ème session.

[3] Dibble HL, Bar-Yosef O (1995). The Definition and Interpretation of Levallois Technology.

[4] Tryon CA (2006). ‘Early’ Middle Stone Age lithic technology of the Kapthurin Formation (Kenya).. Curr Anthropol.

[5] Morgan LE, Renne PR (2008). Diachronous dawn of Africa's Middle Stone Age: New ^40^AR/^39^AR ages from the Ethiopian Rift.. Geology.

[6] Moncel M-H, Moigne A-M, Sam Y, Combier J (2011). The emergence of Neanderthal technical behavior: new evidence from Orgnac 3 (Level 1, MIS 8), southeastern France.. Curr Anthropol.

[7] Porat N, Chazan M, Schwarcz H, Horwitz LK (2002). Timing of the Lower to Middle Paleolithic boundary: new dates from the Levant.. J Hum Evol.

[8] Stringer C (2002). Modern human origins: progress and prospects.. Philos T R Soc B.

[9] Hublin J-J (2009). The origin of Neandertals.. P Natl Acad Sci USA.

[10] Mellars P (1996). The Neanderthal Legacy: An Archaeological Perspective from Western Europe.

[11] Lieberman P (1975). On the Origins of Language: An Introduction to the Evolution of Speech.

[12] Schlanger N (1996). Understanding Levallois: lithic technology and cognitive archaeology.. Camb Archaeological J.

[13] Noble W, Davidson I (1996). Human Evolution, Language and Mind: A Psychological and Archaeological Enquiry.

[14] Wynn T, Coolidge FL (2004). The expert Neandertal mind.. J Hum Evol.

[15] Hayden B (1993). The cultural capacities of Neandertals: a review and re-evaluation.. J Hum Evol.

[16] Bordes F (1968). The Old Stone Age.

[17] Van Peer P (1992). The Levallois Reduction Strategy.

[18] Bordes F (1950). Principes d'une method d'étude des techniques de debitage et de la typologie du Paléolithique acient et moyen.. L'Anthropologie.

[19] Bordes F (1980). Le débitage Levallois et ses variants.. Bull Soc Préhist Fr.

[20] Chazan M (1997). Redefining Levallois.. J Hum Evol.

[21] Brantingham PJ, Kuhn SL (2001). Constraints on Levallois core technology: a mathematical model.. J Archaeol Sci.

[22] Inizan M-L, Reduron-Ballinger M, Roche H, Tixier J (1999). Technology and Terminology of Knapped Stone.

[23] Boëda E, Kozlowski J (1988). Le concept laminare: rupture et filiation avec le concept Levallois.. L'Homme Neanderthal, vol. 8, La Mutation.

[24] Boëda E (1994). Le Concept Levallois: Variabilité des Méthodes.

[25] Boëda E, Dibble HL, Bar-Yosef O (1995). Levallois: a volumetric construction, methods, a technique.. The Definition and Interpretation of Levallois Technology.

[26] Delagnes A, Dibble HL, Bar-Yosef O (1995). Variability within uniformity: three levels of variability within the Levallois system.. The Definition and Interpretation of Levallois Technology.

[27] Ambrose SH (2001). Paleolithic technology and human evolution.. Science.

[28] Klein RG (2009). The Human Career: Human Biological and Cultural Origins, Third Edition.

[29] Pelegrin J, Roux V, Bril B (2005). Remarks about archaeological techniques and methods of knapping: elements of a cognitive approach to stone knapping.. Stone Knapping: the Necessary Conditions for a Uniquely Hominin Behaviour.

[30] Holloway RL (1969). Culture: a human domain.. Curr Anthropol.

[31] Lieberman P (1984). The Biology and Evolution of Language.

[32] Sandgathe DM (2004). Alternative interpretation of the Levallois reduction technique.. Lithic Technology.

[33] Davidson I, Noble W, Gibson KR, Ingold T (1993). Tools and language in human evolution.. Tools, Language and Cognition in Human Evolution.

[34] Eren MI, Bradley B (2009). Experimental evaluation of the Levallois “core shape maintenance” hypothesis.. Lithic Technology.

[35] Dibble HL, Mellars P, Stringer C (1989). The implications of stone tool types for the presence of language during the Lower and Middle Palaeolithic.. The Human Revolution: Behavioural and Biological Perspectives on the Origins of Modern Humans.

[36] Pepère M (1986). Apport de la typométrie à la definition des éclats Levallois.. Bull Soc Préhist Fr.

[37] Kuhn SL, Nowell A, Davidson I (2010). On standardization in the Paleolithic: measures, causes and interpretations of metric similarity in stones tools.. Stone Tools and the Evolution of Human Cognition.

[38] Pittman JR, Ireland HA (1959). Silica in Edwards limestone, Travis County, Texas.. Silica in Sediments: A Symposium with Discussions.

[39] Bradley B (1977). Experimental Lithic Technology with Special Reference to the Middle Palaeolithic..

[40] Prasciunas MM (2007). Bifacial cores and flake production efficiency: an experimental test of technological assumptions.. Am Antiquity.

[41] Shennan S (1997). Quantifying Archaeology.

[42] Hair JF, Anderson RE, Tatham RL, Black WC (1998). Multivariate Data Analysis.

[43] Lycett SJ, von Cramon-Taubadel N, Gowlett JAJ (2010). A comparative 3D geometric morphometric analysis of Victoria West cores: implications for the origins of Levallois technology.. JArchaeol Sci.

[44] Jungers WL, Falsetti AB, Wall CE (1995). Shape, relative size, and size adjustments in morphometrics.. Yearb Phys Anthropol.

[45] Lycett SJ, von Cramon-Taubadel N, Foley RA (2006). A crossbeam co-ordinate caliper for the morphometric analysis of lithic nuclei: a description, test and empirical examples of application.. J Archaeol Sci.

[46] Kelly RL (1995). The Foraging Spectrum: Diversity in Hunter-Gatherer Lifeways.

[47] Binford LR (1980). Willow smoke and dog's tails: hunter-gatherer settlement systems and archaeological site formation.. Am Antiquity.

[48] Geneste J-M, Otte M (1989). Economie des resources lithiques dans le Moustérien du sud-ouest de la France.. L'Homme de Neanderthal, vol. 6, La Subsistence.

[49] Féblot-Augustins J, Roebroeks W, Gamble C (1999). Raw material transport patterns and settlement systems in the European Lower and Middle Palaeolithic: continuity, change and variability.. The Middle Palaeolithic Occupation of Europe.

[50] Roebroeks W, Kolen J, Rensink E (1988). Planning depth, anticipation and the organization of Middle Palaeolithic technology: the ‘Archaic Natives’ meet Eve's descendants.. Helinium.

[51] McBrearty S, Brooks AS (2000). The revolution that wasn't: a new interpretation of the origin of modern human behaviour.. J Hum Evol.

[52] White MJ, Pettitt PB (1995). Technology of early Palaeolithic western Europe: innovation, variability and a unified framework.. Lithics.

[53] Kuhn SL (1994). A formal approach to the design and assembly of mobile toolkits.. Am Antiquity.

[54] Key A, Lycett SJ (2011). Technology based evolution? A biometric test of the effects of ha1ndsize versus tool form on efficiency in an experimental cutting task.. J Archaeol Sci.

[55] Rolian C, Lieberman DE, Zermeno JP (2011). Hand biomechanics during simulated stone tool use.. J Hum Evol.

[56] Surovell TA (2009). Toward a Behavioral Ecology of Lithic Technology: Cases from Paleoindian Archaeology.

[57] Braun DR, Pobiner BL, Thompson JC (2008). An experimental investigation of cut mark production and stone tool attrition.. J Archaeol Sci.

[58] Braun DR, Plummer T, Ferraro JV, Ditchfield P, Bishop LC (2009). Raw material quality and Oldowan hominin toolstone preferences: evidence from Kanjera South, Kenya.. J Archaeol Sci.

[59] Turq A, Dibble H, Mellars P (1992). Raw material and technological studies of the Quina Mousterian in Perigord.. The Middle Paleolithic: Adaptation, Behavior, and Variability.

[60] Simão J (2002). Tools evolve: the artificial selection and evolution of Paleolithic stone tools.. Behav Brain Sci.

[61] Machin AJ, Hosfield RT, Mithen SJ (2007). Why are some handaxes symmetrical? Testing the influence of handaxe morphology on butchery effectiveness.. J Archaeol Sci.

[62] Shea JJ, Davis Z, Brown K (2001). Experimental tests of Middle Palaeolithic spear points using a calibrated crossbow.. J Archaeol Sci.

[63] Sisk M, Shea J (2009). Experimental use and quantitative performance analysis of triangular flakes (Levallois points) used as arrowheads.. J Archaeol Sci.

[64] Fitch WT (2000). The evolution of speech: a comparative review.. Trends Cogn Sci.

[65] Lieberman DE (2011). The Evolution of the Human Head.

[66] Chomsky N (2006). Language and Mind, Third Edition.

[67] Peresani M, Fiore I, Gala M, Romandini M, Tagliacozzo A (2011). Late Neandertals and the intentional removal of feathers as evidenced from bird bone taphonomy at Fumane Cave 44 ky B.P., Italy.. P Natl Acad Sci USA.

[68] Zilhão J, Angelucci DE, Badal-García E, d'Errico F, Daniel F (2010). Symbolic use of marine shells and mineral pigments by Iberian Neandertals.. P Natl Acad Sci USA.

[69] Powell A, Shennan S, Thomas MG (2009). Late Pleistocene demography and the appearance of modern human behavior.. Science.

