# Supplementary material for: Why Levallois? A Morphometric Comparison of Experimental ‘Preferential’ Levallois Flakes versus Debitage Flakes
Source: PLoS One. 2012 Jan 23;7(1):e29273. doi: 10.1371/journal.pone.0029273 (PMC3264556; doi:10.1371/journal.pone.0029273)
Supplement: Text S1 — A comparative 3D geometric morphometric analysis of the experimental Levallois cores and archaeological examples. (DOC) [file pone.0029273.s001.doc]

**Text S1. A comparative 3D geometric morphometric analysis of the experimental Levallois cores and archaeological examples**

In order to corroborate that the Levallois cores produced for this study replicated archaeological examples of Levallois accurately, we undertook a comparative 3D geometric morphometric analysis. In this analysis, we compared directly the 25 remnant Levallois cores produced during our experiments against a comparative sample of genuine archaeological Levallois cores discovered at nine sites in Africa, western Asia and Europe (comparative sample: *n* = 152 cores). This use of geometric morphometric methods enabled direct comparison of shape properties in the upper (Levallois) surface and plan form (outer extremities) of the experimental Levallois cores against the genuine archaeological examples, all of which conformed to Boëda’s (1994, 1994) ‘volumetric’ definition of Levallois.

*Morphometric Methods*

Geometric morphometric techniques utilize analyses of landmark configurations and are particularly appropriate in comparative studies of shape due to their mathematical ability to separate out the properties of ‘shape’ and ‘size’ (O’Higgins, 2000). ‘Shape’ in this analytical context is defined explicitly as the geometric properties of a specimen excluding the effects of isometric scale (or ‘size’) (Slice, 2005). In this instance, such techniques thus enable a quantitative corroboration that the shapes of the experimentally produced Levallois cores accurately replicate the shape of genuine archaeological examples. Such a proposition may be tested via a multivariate comparative analysis of shape variation. If the experimental Levallois cores fit comfortably within the range of shape variation exhibited by the archaeological examples, this verifies that the experimental objects replicate prehistoric examples with high degrees of accuracy.

For the analysis, a landmark configuration of 51 geometrically defined semilandmarks was employed (see below). Landmark configurations were scaled to the same unit size via generalized Procrustes analysis (GPA), using the freely available software *Morphologika* 2.3.1 (O’Higgins and Jones, 2006). GPA proceeds by reducing all landmark configurations to the same unit size (thus removing variation between specimens due solely to differences in isometric size) via use of centroid size. Centroid size is defined as the square root of the summed squared Euclidean distances from each landmark to the centroid of the landmark configuration (Niewoehner, 2005; Slice 2007). Following adjustments for size, GPA uses a least squares criterion to minimize residual differences remaining between configurations due to translation and rotation (Gower, 1975; Chapman, 1990). All remaining variation between landmark positions (Procrustes residuals) can then be interpreted as shape differences.

Following GPA, *Morphologika* projects the Procrustes residuals into a linear shape space and subjects these residuals to principal components analysis (PCA). PCA enables the major aspects of shape variation between analytical specimens (in this case the experimental and archaeological Levallois cores) to be examined in a hierarchical fashion, and determination of whether the shape of one set of specimens is comparable with that of another set (for general information on PCA see: Hair et al. 1998; Shennan, 1997). The first principal component (PC) describes the primary axis of shape variation between specimens (size already having been controlled for via GPA). The second PC describes the second most dominant aspects of variation, with subsequent PCs explaining sequential smaller aspects of the overall variation.

We used a landmark configuration of 51 geometrically defined co-ordinates (‘semilandmarks’) recorded using a Crossbeam Co-ordinate Caliper (Lycett et al., 2006). Full details of the semilandmarking protocol, orientation of artefacts and definitions of all landmarks can be found in Lycett et al. (2006, 2010).

*Materials: comparative sample of archaeological Levallois cores*

We used a comparative archaeological sample of 152 Levallois cores discovered at nine sites in Africa, western Asia and Europe. Each of these Levallois cores conformed to Boëda’s (1994, 1995) volumetric definition (see main paper). Analyses of Levallois that have utilized this material previously include: Lycett 2007a, 2007b, 2009; Lycett et al., 2010. Specifically, the comparative sample was comprised of material from the following sites:

1. Baker’s Hole (Northfleet), Kent, United Kingdom (*n* = 23 cores)

2. Bezez Cave (Level B), Adlun, Lebanon (*n* = 28 cores)

3. El Arabah, Abydos, Egypt (*n* = 16 cores)

4. El Wad (Level F), Israel (*n* = 27 cores)

5. Fitz James, Oise, France (*n* = 11 cores)

6. Kamagambo, Kenya (*n* = 13 cores)

7. Kharga Oasis (KO6e), Egypt (*n* = 11 cores)

8. Muguruk, Kenya (*n* = 12 cores)

9. Soan Valley, Pakistan (*n* = 11 cores)

*Results and discussion of comparative 3D geometric morphometric analysis*

Figure S1 shows PC1 plotted against PC2. Figures S2 and S3 show (respectively) PC1 plotted against PC3, and PC2 plotted against PC3. Cumulatively, PCs 1-3 explain 56.14% of shape variance. In all three plots of these primary axes, the experimental Levallois cores fit comfortably within the range exhibited by the genuine archaeological examples. This demonstrates unequivocally that the landmark configurations observed for the experimental cores are not atypical when compared against the equivalent landmark configurations of the archaeological Levallois. This analysis thus supports the proposition that the experimental Levallois replicas produced in this study, are close enough to known archaeological examples to be analytically informative.

*References*

Boëda, E., 1994. *Le Concept Levallois: Variabilité des Méthodes*. Centre de la Recherche Scientifique (CNRS), Paris.

Boëda, E., 1995. Levallois: a volumetric construction, methods, a technique. In: H. L. Dibble and O. Bar-Yosef (Eds.), *The Definition and Interpretation of Levallois Technology*, pp. 41-68. Prehistory Press, Madison, Wisconsin.

Chapman, R.E., 1990. Conventional Procrustes approaches. In: Rohlf, F.J., Bookstein, F.L. (Eds.), *Proceedings of the Michigan Morphometrics Workshop Special Publication No. 2*. University of Michigan Museum of Zoology, Ann Arbor, pp. 251–267.

Gower, J.C., 1975. Generalised Procrustes analysis. *Psychometrika* 40, 33–50.

Hair, J.F., Anderson, R.E., Tatham, R.L. and Black, W.C., 1998. *Multivariate Data Analysis*. Prentice-Hall, Upper Saddle River, NJ.

Lycett, S.J., 2007a. Is the Soanian techno-complex a Mode 1 or Mode 3 phenomenon? A morphometric assessment. *Journal of Archaeological Science* 34 (9): 1434-1440.

Lycett, S.J., 2007b. Why is there a lack of Mode 3 Levallois technologies in East Asia? A phylogenetic test of the Movius-Schick hypothesis. *Journal of Anthropological Archaeology* 26 (4): 541-575.

Lycett, S.J., 2009. Are Victoria West cores 'proto-Levallois'? A phylogenetic assessment. *Journal of Human Evolution* 56 (2): 175-191.

Lycett, S.J., von Cramon-Taubadel, N. and Foley, R.A., 2006. A crossbeam co-ordinate caliper for the morphometric analysis of lithic nuclei: a description, test and empirical examples of application. *Journal of Archaeological Science* 33 (6): 847-861.

Lycett, S.J., von Cramon-Taubadel, N. and Gowlett, J.A.J., 2010. A comparative 3D geometric morphometric analysis of Victoria West cores: implications for the origins of Levallois technology. *Journal of Archaeological Science* 37 (5): 1110-1117.

O'Higgins, P., 2000. The study of morphological variation in the hominid fossil record: biology, landmarks and geometry. *Journal of Anatomy* 197: 103-120.

O’Higgins, P. and Jones, N., 2006. *Tools for Statistical Shape Analysis*. Hull York Medical School.

Niewoehner, W.A., 2005. A geometric morphometric analysis of late Pleistocene human metacarpal 1 base shape. In: Slice, D.E. (Ed.), *Modern Morphometrics in Physical Anthropology*. Kluwer, New York, pp. 285–298.

Shennan, S., 1997. *Quantifying Archaeology*. Edinburgh University Press, Edinburgh.

Slice, D.E., 2005. Modern morphometrics. In: Slice, D.E. (Ed.), *Modern Morphometrics in Physical Anthropology*. Kluwer, New York, pp. 1–45.

Slice, D.E., 2007. Geometric morphometrics. *Annual Review of Anthropology* 36: 261-281.
