# Supplementary material for: Why Levallois? A Morphometric Comparison of Experimental ‘Preferential’ Levallois Flakes versus Debitage Flakes
Source: PLoS One. 2012 Jan 23;7(1):e29273. doi: 10.1371/journal.pone.0029273 (PMC3264556; doi:10.1371/journal.pone.0029273)
Supplement: Text S2 — Flake variables measured for analyses. (DOC) [file pone.0029273.s002.doc]

**Text S2. Flake variables measured for analyses**

A total of 15 variables were recorded for each of the 642 flakes used in the analyses. All plan-form flake measurements (i.e. length and width variables 1-6) were measured with digital sliding calipers. All thickness measurements (i.e. variables 7-13) were taken with digital spreading calipers. Variables 1-14 were recorded in mm.

1. Maximum length

Since our primary analyses were directed toward understanding the attributes of flakes as tool/blank forms, our major axis of orientation for measurement was that of absolute maximum dimension (‘length’), with the majority of variables (i.e. 1-5, 7-11) being located in relation to this primary axis. The ‘Maximum length’ of each flake was thus defined as the maximum dimension across the surface of the flake. For remaining measurements, flakes were orientated such that maximum surface area and/or mass was orientated as the proximal (100% of length) end of the flake while that with least surface area and/or mass was defined as the distal (i.e. 0% of length) end of the flake. The dorsal surface of the flake was always treated as the superior (upper) surface. The distal ends of ‘Maximum length’ were marked on each flake with a soft pencil in order to assist with the recording of other variables, as described below.

2. Maximum width

‘Maximum’ width was defined as the maximum dimension of the flake orthogonal to the axis of Maximum length. The distal ends of this measurement were marked on each flake with a soft pencil in order to assist with the recording of other variables, as described below (i.e. variables 10-11).

3. Width at 25% of Maximum length

This variable is defined as the dimension across the width of the flake (orthogonal to Maximum length) at 25% along the Maximum length (with the flake orientated as described above). 1mm^2^ graph paper and marking of each flake with a soft pencil at the appropriate points of the lateral edge of the flake were used to assist this procedure.

4. Width at 50% of Maximum length

This variable is defined as the dimension across the width of the flake (orthogonal to Maximum length) at 50% along the Maximum length (with the flake orientated as described above). 1mm^2^ graph paper and marking of each flake with a soft pencil at the appropriate points of the lateral edge of the flake were used to assist this procedure.

5. Width at 75% of Maximum length

This variable is defined as the dimension across the width of the flake (orthogonal to Maximum length) at 75% along the Maximum length (with the flake orientated as described above). 1mm^2^ graph paper and marking of each flake with a soft pencil at the appropriate points of the lateral edge of the flake were used to assist this procedure.

6. Length of flake (technological)

‘Technological length’ was defined as the dimension of the flake from the point of percussion to the distal edge of the flake with the measurement orientated in the axis of flaking (Debénath and Dibble 1994).

7. Thickness at 25% of Maximum length

This variable is defined as the thickness of the flake at the point of 25% along the Maximum length line. The pencil markings recorded in previous measurements are used to locate this point accurately.

8. Thickness at 50% of Maximum length

This variable is defined as the thickness of the flake at the point of 50% along the Maximum length line. The pencil markings recorded in previous measurements are used to locate this point accurately.

9. Thickness at 75% of Maximum length

This variable is defined as the thickness of the flake at the point of 75% along the Maximum length line. The pencil markings recorded in previous measurements are used to locate this point accurately.

10. Thickness at 25% of Maximum width

This variable is defined as the thickness of the flake at the point of 25% along the Maximum width line. The pencil markings recorded in previous measurements are used to locate this point accurately.

11. Thickness at 75% of Maximum width

This variable is defined as the thickness of the flake at the point of 75% along the Maximum width line. The pencil markings recorded in previous measurements are used to locate this point accurately.

12. Maximum flake thickness

This variable is defined as the maximum dimension of the flake (wherever it may occur) orthogonal to the plane defined by the Maximum length and Maximum width measures.

13. Bulb thickness

This variable is defined as the maximum dimension of the flake across the percussion bulb, taken orthogonally to the plane defined by the technological length.

14. Length of sharp edge

Ethnographic and experimental studies indicate that flake-edge angles of ≤50° provide a common upper limit for functional edges in (unretouched) flake tools (see Prasciunas 2007 and references therein). Hence, we defined the ‘sharp edge’ of each flake according to this threshold of an edge-angle of ≤50°. In ambiguous cases, this edge-angle was verified manually using a goniometer. The measurement is obtained by tracing a piece of nylon string along the sharp edge(s) of the flake (so defined) and then measuring the length of the string. It is important to note that only a small portion of the string is required to be in contact with the flake at any one time, if the measurement is taken from a mark that is made on the string as a start point. This measurement is a variation of the technique described by Braun (2005, pp. 112–113).

15. Index of symmetry

An ‘Index of Symmetry’ may be calculated as the summed differences between a series of bilateral measurements, corrected for absolute size (Lycett 2008). The pencil markings produced during the recording of previous measurements and graph paper were used to calculate the bilateral distances (mm) from the line of ‘Maximum length’ to the lateral edges of the flake at the 25%, 50% and 75% points.

The Index of Symmetry (*S*) is then computed as:

$$S=\sum_{i=1}^{n} \left(\frac{\sqrt{(Xi-Yi)^{2}}}{Xi+Yi}\right)$$

Where, *X_i_* is the width value taken to the left of maximum length at one of the percentage points (i.e. 25, 50, 75%) along that length. *Y_i_* is the corresponding width value right of the maximum length line, and *n* is the number of percentage points taken (in this case three). Hence, a value of zero would correspond to perfect symmetry, with values increasing as the form moves further away from symmetry. It is important to note that *S* is expressed as a ratio of the degree of difference between the two lateral measurements (*X_i_* – *Y_i_* ) and overall width at that percentage point (*X_i_* + *Y_i_* ), ensuring that *S* is a scale-free (size-independent) measure.

**References**

Braun, D.R., 2005. Examining flake production strategies: examples from the Middle Paleolithic of southwest Asia. *Lithic Technology* 30: 107-125.

Debénath, A. and Dibble, H.L., 1994. *Handbook of Paleolithic Typology*. University Museum, University of Pennsylvania, Philadelphia.

Lycett, S.J., 2008. Acheulean variation and selection: does handaxe symmetry fit neutral expectations? *Journal of Archaeological Science* 35 (9): 2640-2648.

Prasciunas, M.M., 2007. Bifacial cores and flake production efficiency: an experimental test of technological assumptions. *American Antiquity* 72 (2): 334-348.
