# Supplementary figures and images for: Why Levallois? A Morphometric Comparison of Experimental ‘Preferential’ Levallois Flakes versus Debitage Flakes
Source: PLoS One. 2012 Jan 23;7(1):e29273. doi: 10.1371/journal.pone.0029273 (PMC3264556; doi:10.1371/journal.pone.0029273)

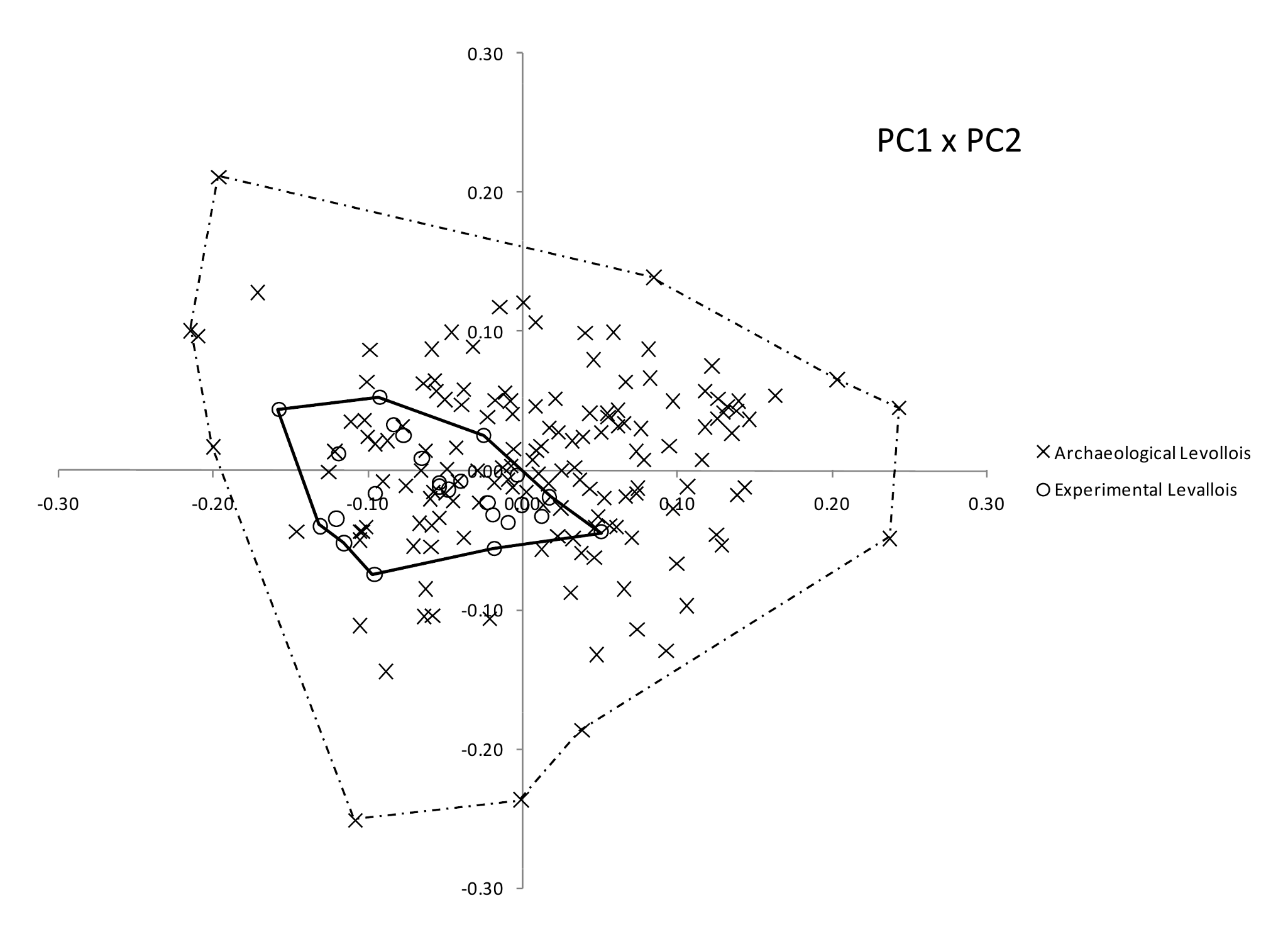

Supplement: Figure S1 — Principal component results of 3D geometric morphometric analysis comparing archaeological examples of Levallois core against the experimental replicas produced for this study. This Figure shows PC1 plotted against PC2. (TIF) [file pone.0029273.s003.tif]

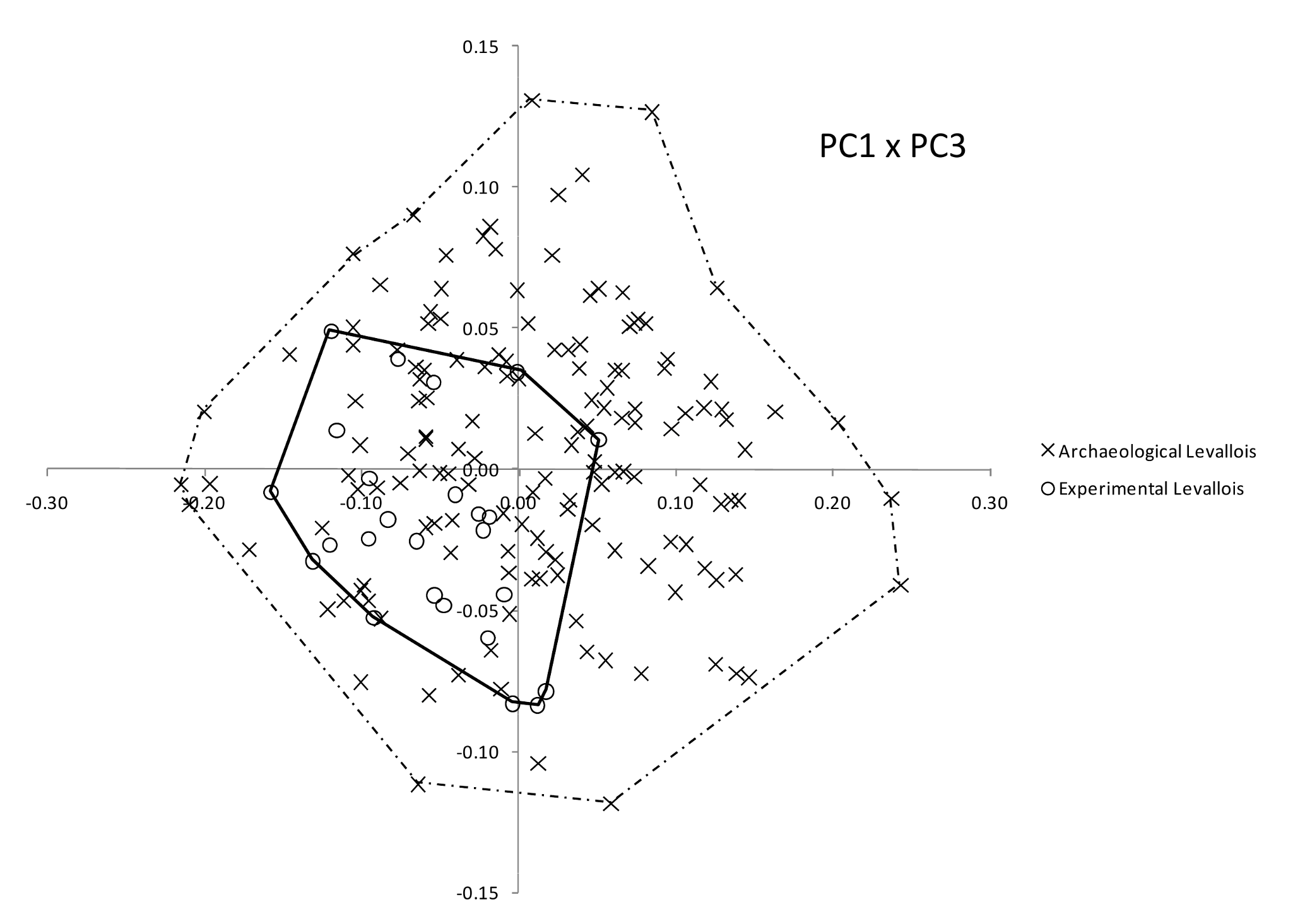

Supplement: Figure S2 — Principal component results of 3D geometric morphometric analysis comparing archaeological examples of Levallois core against the experimental replicas produced for this study. This Figure shows PC1 plotted against PC3. (TIF) [file pone.0029273.s004.tif]

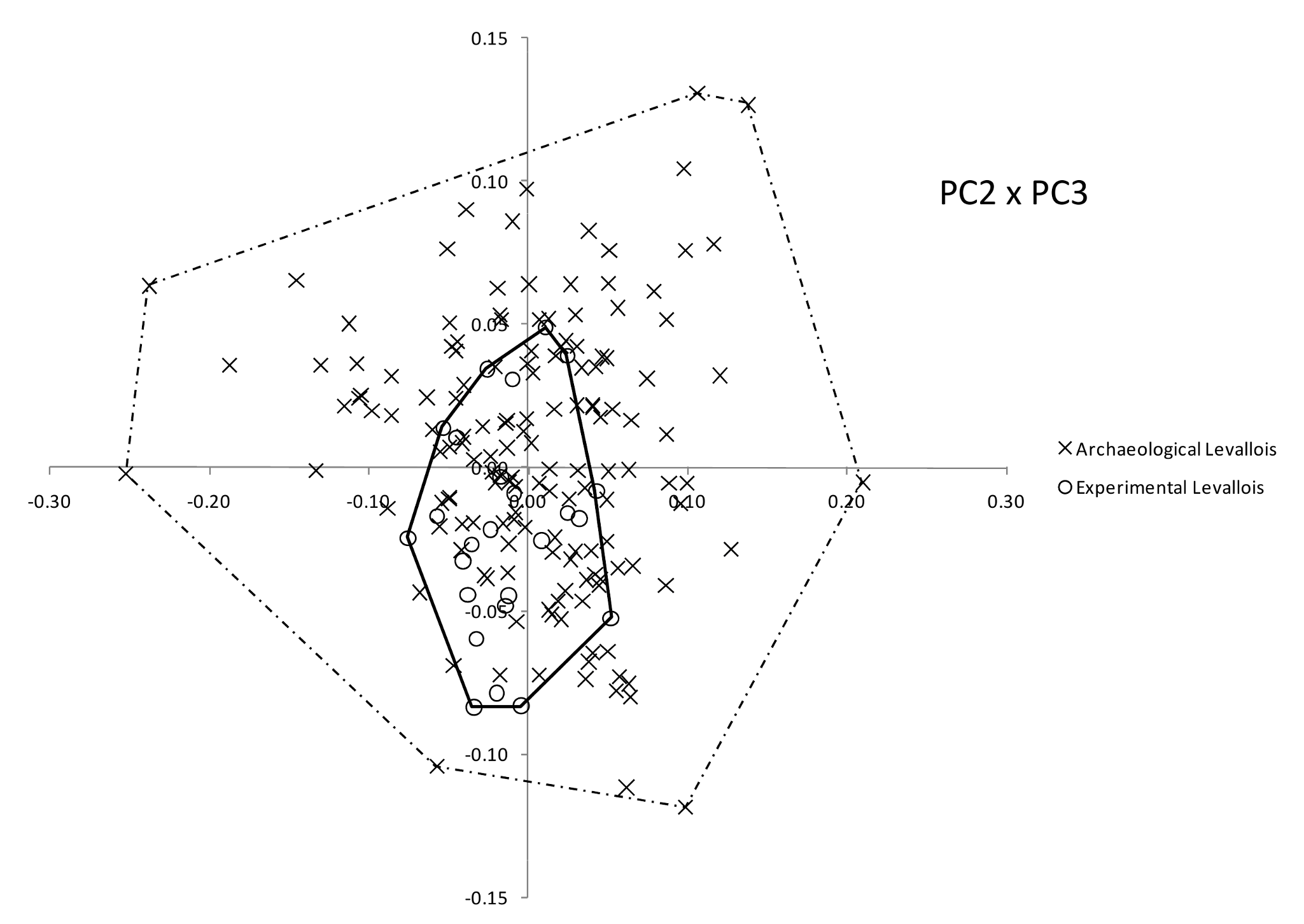

Supplement: Figure S3 — Principal component results of 3D geometric morphometric analysis comparing archaeological examples of Levallois core against the experimental replicas produced for this study. This Figure shows PC2 plotted against PC3. (TIF) [file pone.0029273.s005.tif]
